# Serum-derived host miR-423-5p and miR-378a-3p as molecular markers for severe tuberculosis: a promising prognostic tool for survival

**DOI:** 10.3389/ftubr.2024.1441258

**Published:** 2024-11-29

**Authors:** Joaquin Hurtado, María Buroni, Alvaro Giordano, Nicolas Nin, F. Javier Hurtado, Juan Pablo Tosar, Carlos Robello, Gonzalo Greif

**Affiliations:** ^1^Laboratorio de Interacciones Hospedero-Patógeno, Institut Pasteur de Montevideo, Montevideo, Uruguay; ^2^Unidad de Cuidados Intensivos, Hospital Español Dr. “Juan J. Crottoggini”, Administración de los Servicios de Salud del Estado (ASSE), Montevideo, Uruguay; ^3^Departamento de Fisiopatología, Facultad de Medicina, Universidad de la República, Montevideo, Uruguay; ^4^Laboratorio de Genómica Funcional, Institut Pasteur de Montevideo, Montevideo, Uruguay; ^5^Unidad de Bioquímica Analítica, Centro de Investigaciones Nucleares, Facultad de Ciencias, Universidad de la República, Montevideo, Uruguay; ^6^Departamento de Bioquímica, Facultad de Medicina, Universidad de la República, Montevideo, Uruguay

**Keywords:** *Mycobacterium tuberculosis*, tuberculosis, critical care medicine, small RNA, miRNA

## Abstract

Tuberculosis (TB) remains a leading cause of infectious disease-related mortality. Annually, 10 million people contract TB, resulting in 1.5 million deaths, despite being a preventable and curable disease. Severe TB cases necessitate Intensive Care Unit (ICU) admission, with mortality rates ranging from 15.5 to 65.9%. Recent research highlights the role of microRNAs (miRNAs) in infectious disease diagnosis, with studies reporting distinct miRNA profiles in active pulmonary TB and sputum samples. This study aims to identify miRNAs as potential prognostic biomarkers for severe TB in ICU patients. Total RNA was extracted from the serum of ICU TB patients and controls. miRNA libraries were prepared and high throughput sequenced using a MiSeq Illumina platform. Differential miRNA abundance between patients and controls was analyzed with sRNAtoolbox, and DESeq2 was used for comparisons. Results demonstrated three differentially abundant miRNAs in severe TB patients' serum, validated by RT-qPCR. Stratifying patients by outcome revealed a significant difference in the ratio between two miRNAs: hsa-miR-378a-3p and hsa-miR-423-5p. The analysis showed that a miRNA-423-5p/miRNA-378a-3p ratio < 27 is associated with a poor prognosis, highlighting its potential as a prognostic indicator of disease severity. These findings are promising and warrant validation, while assessing these biomarkers in non-severe TB settings could further help identify more aggressive forms of the disease. In conclusion, this study explores the potential of circulating miRNAs as prognostic tools for severe TB cases in the ICU, offering a promising avenue for improving clinical decision-making and patient outcomes. Further validation and exploration in diverse TB contexts are essential for comprehensive understanding and application.

## Introduction

Tuberculosis disease still represents the primary cause of death by an infectious agent (2023 WHO Tuberculosis Global Report), second only to COVID-19 pandemic due to SARS-CoV-2 virus between 2020 and 2022. Every year, ~10 million people fall ill with tuberculosis (TB) and despite being a preventable and curable disease, 1.5 million people die (2023 WHO Tuberculosis Global Report). In Uruguay, the mean incidence between 2010 and 2018 was 27 cases per 100,000 inhabitants, with a population of ~3.5 million.

Severe tuberculosis cases need admission to the Intensive Care Unit (ICU) for advanced vital support. Despite the availability of effective therapies, mortality rates in critical care patients range from 15.5 to 65.9% (1, 2). In a previous study, we examined a cohort of ICU patients in Uruguay in the period 2010–2018 with a mortality rate of around 50% (3). During this period, ~1.3% (*n* = 104) of the total TB patients (*n* = 7,560) in the country, required ICU admission. These patients represented 1.6% of the total cases admitted to the ICU during the period. The mean age of these patients was 42 ± 15 years, and males were more frequent (69%). The APACHE II score at admission was 19 ± 10 (3).

Despite advances in tuberculosis (TB) diagnosis, strain characterization, and the determination of resistant profiles, there is currently a lack of prognostic tools for patients in this context. These tools are essential to aid clinicians in making informed treatment and management decisions, ultimately aiming to mitigate the high mortality rates associated with severe TB cases in an ICU context.

Recent studies have highlighted the significance of miRNAs in diagnosing infectious diseases (4–6). An initial investigation into circulating miRNAs in individuals with active pulmonary TB revealed 59 upregulated and 33 downregulated miRNAs compared to non-TB controls (7). Additionally, another study identified the overexpression of miR-3179 and miR-147 in the sputum of TB-infected patients as opposed to controls (8).

Moreover, emerging evidence suggests that miRNAs derived from serum exhibit remarkable stability even under harsh conditions, rendering them valuable as biomarkers for the early diagnosis of various infectious diseases (9).

The objective of this study was to identify miRNAs as potential prognostic biomarkers for severe tuberculosis disease in patients admitted to the Intensive Care Unit (ICU). Small RNA sequencing was conducted on host serum samples obtained from critically ill TB patients and compared with circulating miRNAs from healthy blood donors with sex and agre range composition in range with TB patients. Prospectively, we compared the miRNA profile of ICU survivors and deceased patients, and through this analysis, we successfully identified and validated two circulating miRNAs demonstrating potential prognostic value.

## Materials and methods

### Ethics statement

The study was approved by the Ethics of Investigation Committee of the Hospital Español (Montevideo, Uruguay). Patients older than 18 years old who had Pulmonary, or Extrapulmonary TB diagnosis before or during the ICU stay were included. The study period was from 01/01/2010 to 12/31/2018.

### Patients

A clinical descriptive analysis was made among critically ill patients in a general Intensive Care Unit, located at the Hospital Español in Montevideo, Uruguay.

The diagnosis of TB was suspected based on compatible clinical and radiologic findings in the context of suggestive epidemiologic information. The diagnostic confirmation was assumed when one or more of the following data were present: (a) Positive culture for *Mycobacterium tuberculosis*; (b) Acid-Alcohol Resistant Bacilli in sputum or bronchoalveolar lavage; (c) Positive GeneXpert for *Mycobacterium tuberculosis*; (d) histopathological findings in tissue autopsy/biopsy when corresponding.

Demographic information included comorbid and socioeconomic conditions were recorded. Particular clinical presentations such as hemoptysis, pneumothorax, atelectasis, pleural effusions, and were also documented. Laboratory and physiologic parameters at admission to the ICU, presence of organ dysfunction and APACHE II scores were recorded. The principal outcomes registered were days on mechanical ventilation, length of stay, and ICU mortality (3).

Serum of all patients enrolled in the study were obtained at the entry to the ICU. Briefly, 5 mL of peripheral blood were obtained in serum collection tubes. The tubes were immediately centrifuged at 1,000 *g* for 10 min, and the serum was recovered in 1.5 mL Eppendorf tubes and frozen at −80°C within 1 h after blood collection. All serum samples were processed uniformly. This approach was adopted to avoid possible variations in miRNA abundance attributed to differences in processing. Only non-hemolyzed sera were used.

Summarized data (age, gender, Apache II, risk factors, days at ICU, outcome, and TB strain) from all patients enrolled as well as control blood donors were included in Supplementary Table 1.

ICU patients were segregated into two groups based on outcome: TB survivors (patients who no longer required ICU support) and TB deceased patients (those who unfortunately died during their ICU stay). Additionally, the survivors were alive after ICU discharge, and follow-up data confirmed that they remained alive during the 1st year after completing their hospital stay.

### RNA extraction and sequencing

Total RNA extraction (including miRNAs) from serum was performed using the “Quick-cfRNA Serum and Plasma” kit from ZYMO Research (USA) following the manufacturer's instructions. In all cases, 1 mL of serum was used as the starting point.

For small RNA sequencing, libraries were prepared using the “NEBNext small RNA Library Prep Set for Illumina” kit from New England BioLabs (USA). Library quantification was carried out with the Qubit “High Sensitivity dsDNA Assay” kit (Thermo, USA). Sequencing was performed with a “MiSeq Reagent Kit v3” (Illumina, USA) cartridge using a 50-cycle configuration on the MiSeq sequencer available at the Institut Pasteur de Montevideo.

### RT-qPCR

Validation of miRNAs was carried out through amplification using the stem-loop RT-qPCR technique (10) and analysis of relative abundances was performed using the ΔΔCt method, using the hsa-miR-let7b gene for normalization. The hsa-miR-let7b were selected after evaluate reference candidate genes according to Schwarzenbach et al. (11). Primers and cycle conditions are summarized in Supplementary Table 2.

### Bioinformatics

Data analysis involved processing the obtained sequences with the sRNAtoolbox online program package (https://arn.ugr.es/srnatoolbox) (12). Two main programs from this package were primarily used: sRNAbench to observe the abundance profile of small RNAs in each sample and sRNAde for detecting differentially abundant small RNAs among sample groups. DESeq2 were employed for normalization and differential analysis, calculating the fold change and dispersion of these data. The data were mapped against the human genome GRCh38-p13 and miRNA database miRBase release 22.1 was used. The final result tables indicate the Benjamini-Hochberg adjusted *p*-value, and in this analysis, an adjusted *p*-value < 0.05 was considered a significant change (Supplementary Table 3).

Based on the information obtained from the differential abundance analysis, miRNAs with a significant adjusted *p*-value and a high read count (greater abundance compared to blood donors control samples) were proposed as candidates for disease markers or severity prognosis. We used the stats package in Rstudio to perform the Wilcoxon test and calculate *p*-values for comparisons of miRNA abundance between conditions.

R Studio was used to generate boxplots of miRNA abundance. The pROC package was used to calculate the optimal cut-off to discriminate between survival and deceased patients based on the hsa-miR-423-5p and hsa-miR-378a-3p ratio, using the 'best' method which maximizes the Youden index.

## Results

### Sequencing statistics

TB patients were segregated by outcome into a survivor group, with a mean ICU stay of 4.2 days and at least 1 year of follow-up, and a deceased group, who died during their ICU stay, with a mean duration of 15.3 days. We obtained serum total RNA from 13 severe TB patients (eight deceased in the ICU and five survivors) as well as serum from five blood donors used as control samples.

Sequencing results are summarized in Supplementary Table 4. All data is publicly available at Sequence Read Archive (SRA) from NCBI under project, Accession number: PRJNA1084803.

### Differential miRNA profiling

The number of mature miRNAs detected in TB patients was similar between deceased (mean 269 miRNAs) and survival patients (mean 250 miRNAs), both of which were significantly lower compared to the control serum (mean 570 miRNAs, *p*-value < 0.05; Supplementary Figure 1).

Using DESeq2, we then compared the differential abundance of miRNAs between TB patients (deceased and survival combined) and controls. The clustering of samples based on miRNA abundance profiles, successfully classified the samples according to their respective groups (Supplementary Figure 2). This analysis identified 41 miRNAs that were differentialy abundant between patients and controls (logFC>|1.5| and adjusted *p*-value < 0.05; Supplementary Table 3); 13 were more abundant in patients, while 28 were more abundant in controls. From this analysis, three miRNAs were selected for further analysis due to their low intra-group dispersion. Among them, two (hsa-miR-378a-3p and hsa-miR-423-5p) were found to be more abundant in the patient samples, while one (hsa-miR-126-3p) was less abundant (Table 1).

**Table 1:** Differential miRNAs.

**miRNA**	**Controls mean**	**Tb means**	**Fold change**	**Log2-fold change**	***p*-adj**
hsa-miR-126-3p	4,430.70	721.25	6.14	2.60	0.42 × 10^−4^
hsa-miR-378a-3p	117.09	3,345.76	0.03	−1.78	0.11 × 10^−2^
hsa-miR-423-5p	1,490.50	11,159.95	0.12	−2.97	0.42 × 10^−3^

The table summarize the abundance of three differential miRNAs between TB patients (n = 13) and controls (n = 5). Two decimal digits were used.

**Figure 1:**
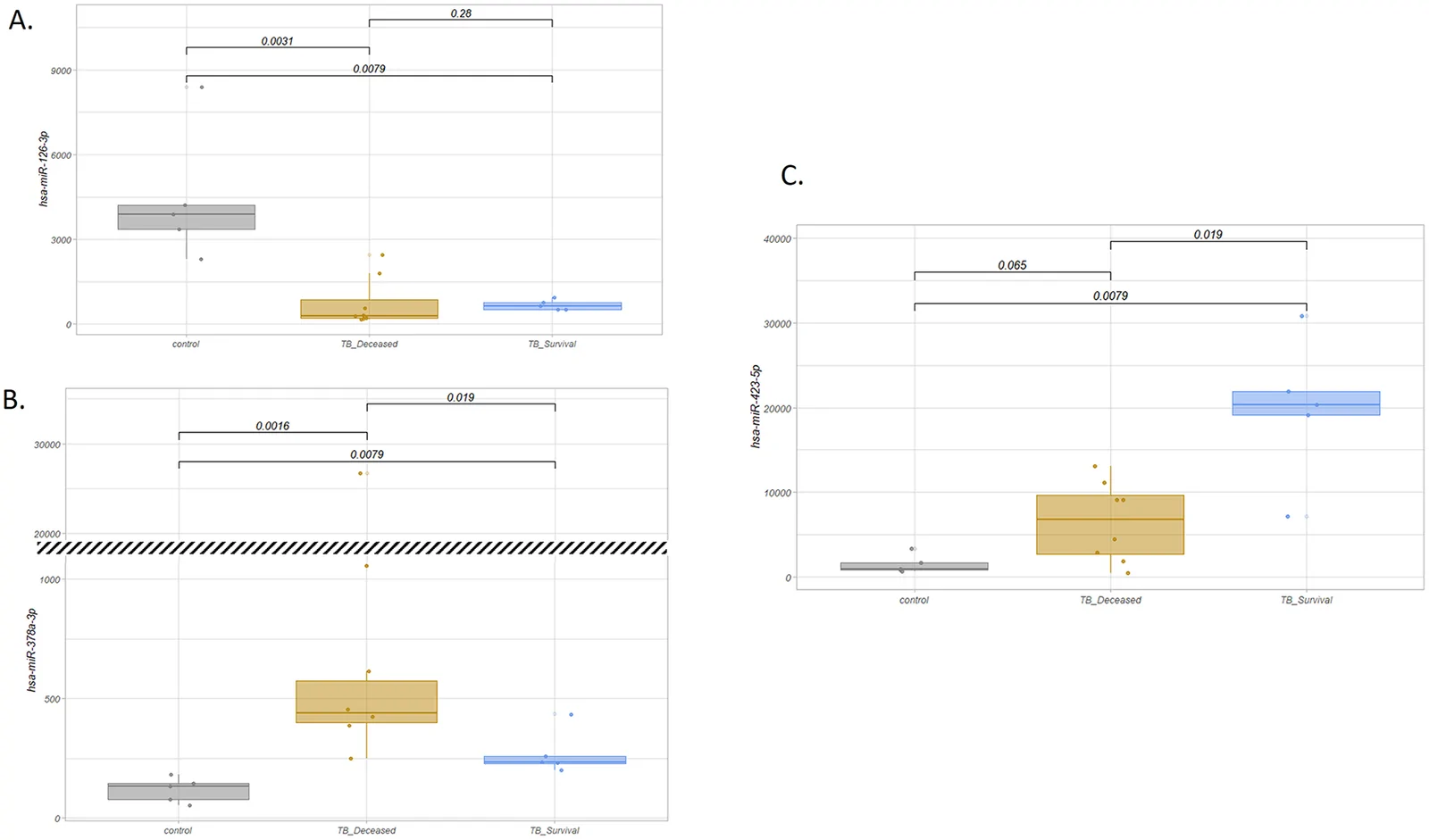
Boxplot of normalized count of selected miRNA by sample type. **(A)** hsa-miR-126-3p: more abundant in control samples (*p*-value = 0.0079 for control vs. survival patients and *p*-value = 0.0031 for control vs. deceased patients comparisons) and with no significant differences between TB subgroups (*p*-value = 0.28). **(B)** hsa-miR-378a-3p: more abundant in TB patients compared to controls (statistically significant differences with *p*-value = 0.0079 and *p*-value = 0.0016 for the comparisons of control vs. survival and control vs. deceased patients, respectively), and opposite to hsa-miR-423-5p, more abundant in deceased vs. survival patients (*p*-value = 0.019). The break in the plot indicates a change in scale. **(C)** hsa-miR-423-5p: more abundant in TB patients compared to controls and in TB survivors vs. deceased patients. *p*-values show statistical differences between TB survivors vs. deceased patients (*p*-value = 0.019).

**Figure 2:**
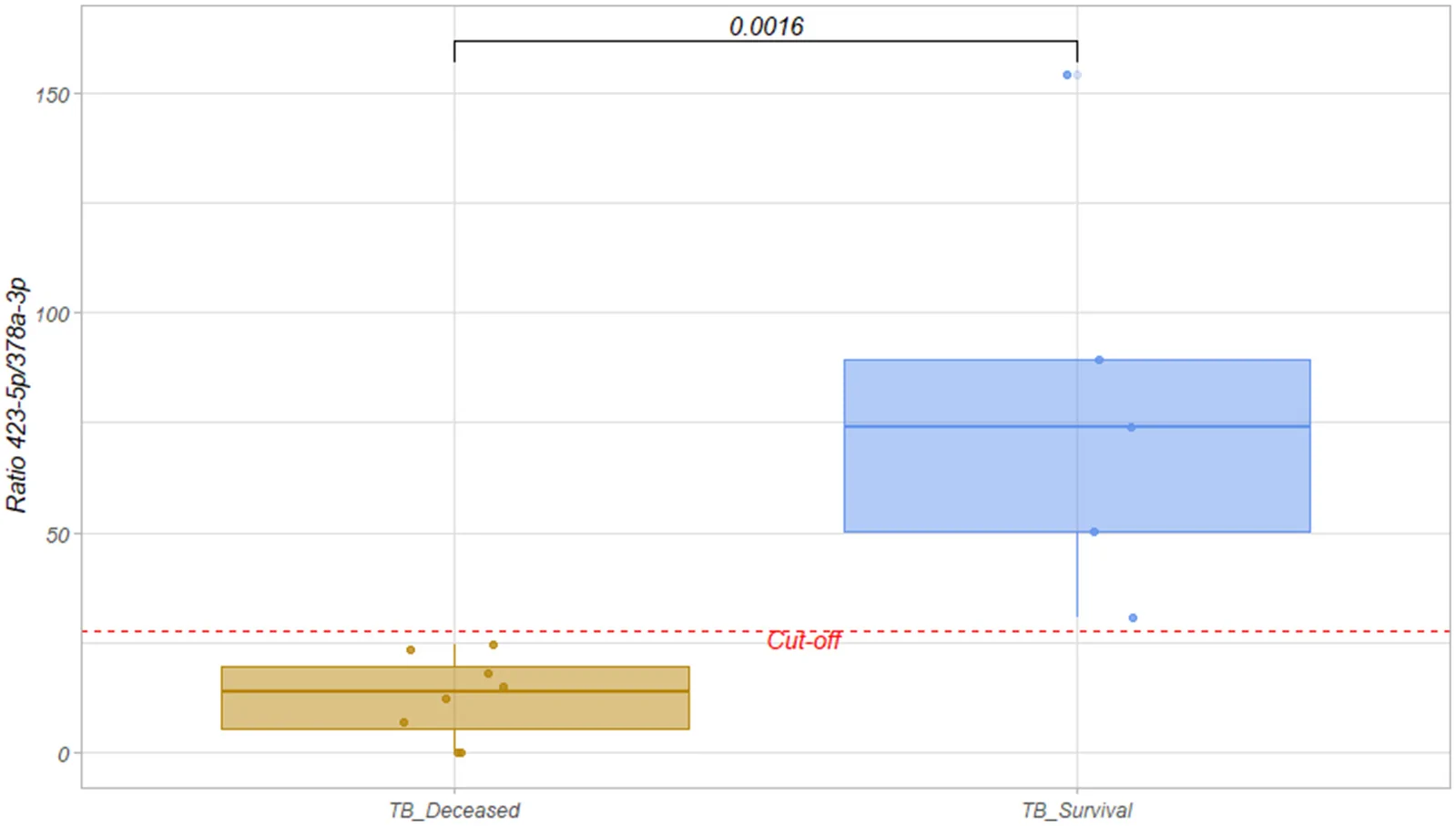
Boxplot of hsa-miR-423-5p/hsa-miR-378a-3p ratio for TB survival (blue) and deceased groups (orange). The ratio was calculated by dividing the abundance of hsa-miR-423-5p by the abundance of hsa-miR-378a-3p in each sample. *Wilcox* test was performed and statistical significance *p*-value = 0.0016 was obtained. The dashed red line indicates the cut-off obtained from the ROC curve, which maximizes the Youden index. Cut-off = 27.63.

In a subsequent analysis, we segregated patients into those who survived and those who later died in the ICU to identify potential prognostic biomarkers. Stratifying the patients by outcome, we observed statistically significant differences between these two groups of patients in the two miRNA previous characterized (Figures 1A–C). Indeed, the ratio of abundance of miR-423-5p (more abundant in survivors) and miR-378a-3p (more abundant in deceased patients) did achieve statistical significance (mean FC ratio = 6.34; *p*-value < 0.0016; Figure 2).

### RT-qPCR validation

To validate the results, we performed stem-loop RT-qPCR for the selected miRNA using RNA purified from the serum of five survival and five deceased patients. miR-423-5p could not be validated due to the limitation of the stem-loop RT-qPCR method in amplifying some sequences. The validation results for miR-378a-3p are shown as relative abundance levels using hsa-miR-let7b as a normalizer (Supplementary Figure 3A). This miRNA was selected as a reference because it showed no variation between patient's groups in our sequencing data (Supplementary Figure 3B).

## Discussion

Tuberculosis infection leading to a critically ill condition occurs as a very advanced stage of the disease. These patients usually carry different comorbidities, need more invasive vital support therapies, and are exposed to frequent complications during ICU stay (3). A significant challenge for clinical professionals is obtaining information about the prospective severity of patients upon admission to the ICU to ensure appropriate management.

In our cohort, we could not link a specific TB lineage to explain the severity or outcome of the disease after entry into the ICU, although we did observe differences in the composition of strains circulating in the general population and those resulting in severe cases requiring ICU admission, with the Haarlem strain being the most frequently found in the latter group (3). In our quest for new tools that can provide information about disease progression and serve as a decision-making tool for clinicians, we opted to analyze the circulating small RNA profile in the serum of these patients and compare it with that of healthy blood donor controls.

It is known that free (13) or protein-associated (14) miRNAs can be detected in serum, and have been demonstrated to be stable molecules that can be used as biomarkers in numerous diseases (6, 15–20). In this context, several miRNAs have been found in patients with acute respiratory distress syndrome (ARDS) and sepsis. For instance, hsa-miR-27, hsa-miR-146, hsa-miR-155-5p, and hsa-miR-223, show elevated blood levels in ARDS patients compared to healthy donors. None of these miRNAs show statistical differences in our study. Furthermore, in septic patients, hsa-miR-223 levels are decreased in non-survivors compared to survivors (21). In our data, hsa-miR-223-5p is higher in TB patients compared to controls, but without significant differences between survivors and deceased patients. One recent study found that hsa-miR-155-5p is one of the most important miRNAs modulating the immune response in tuberculosis infection and is downregulated in patients with active TB infection compared to latent and/or healthy individuals (22). In our data, although hsa-miR-155-5p is downregulated in patients, it does not show statistically significant differences compared to healthy controls. Finally, it is important to highlight that there are also other studies that have established the optimal combination of miRNA ratios as predictors of mortality during ICU stay (23).

From our results, we identified three differentially abundant miRNAs circulating in the serum, two of which were significantly more abundant in the patient samples. In the case of hsa-miR-378a-3p, the result was validated by RT-qPCR. One limitation encountered in the study was the constrained capacity of stem-loop RT-qPCR to validate all of the selected candidate miRNAs.

Upon segregating patient samples into favorable (survivor group) and unfavorable outcome groups (deceased during ICU stay), we observed that the ratio between hsa-miR-423-5p and hsa-miR-378a-3p, two of the identified miRNAs, can serve as a valuable prognostic tool for clinicians, with ratios below 27.63 being a bad prognostic factor. Despite the limitation of processing a small number of samples in this study, due to the low prevalence of TB infection in a country with a mandatory vaccination program, the presented results are promising and warrant further validation. The evaluation of these markers in other non-TB-related critical illnesses is necessary to determine whether they are specific to TB. The data generated includes other circulating small RNA molecules (tRNAs, circRNAs) have also been proposed as potential disease biomarkers (24–31). Furthermore, evaluating these potential biomarkers in non-severe tuberculosis settings could serve as a valuable tool to identify potentially more aggressive forms of the disease.

## Data Availability

The data presented in the study are deposited in the Sequence Read Archive (SRA) repository https://www.ncbi.nlm.nih.gov/sra, accession number PRJNA1084803.
